# *QuickStats:* Birth Rates* by Urbanization Level^†^ and Age Group of Mother — National Vital Statistics System, United States, 2017

**DOI:** 10.15585/mmwr.mm6745a9

**Published:** 2018-11-16

**Figure:**
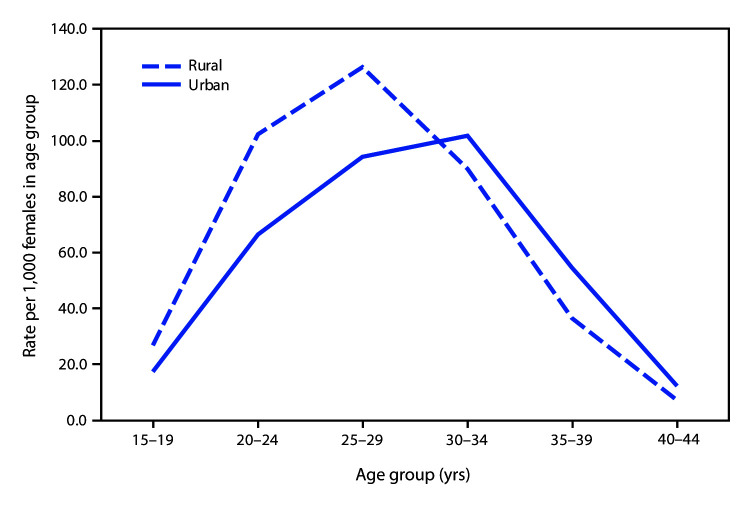
In 2017, women aged <30 years in rural counties had higher birth rates than in urban counties. For women aged ≥30 years, birth rates were higher in urban counties than in rural counties. In 2017, the highest birth rates in rural counties were to women aged 25–29 years (126.4 births per 1,000 women); in urban counties the highest birth rates were to women aged 30–34 years (101.7 births per 1,000 women).

**Source:** NCHS Data Brief no. 323. Trends in fertility and mother’s age at first birth among rural and metropolitan counties: United States, 2007–2017. https://www.cdc.gov/nchs/data/databriefs/db323-h.pdf.

